# The Impact of Hyponatremia on the Severity of Childhood Tuberculous Meningitis

**DOI:** 10.3389/fneur.2021.703352

**Published:** 2022-01-05

**Authors:** Rashid Salih, Ronald van Toorn, James A. Seddon, Regan S. Solomons

**Affiliations:** ^1^Department of Paediatrics and Child Health, Faculty of Medicine and Health Sciences, Stellenbosch University, Cape Town, South Africa; ^2^Department of Infectious Diseases, Imperial College London, London, United Kingdom

**Keywords:** pediatric tuberculous meningitis, hyponatremia, hypoglycorrhachia, outcome, neuro-morbidity

## Abstract

**Introduction:** Hyponatremia and/or hypoglycorrhachia are commonly encountered biochemical derangements during the acute stage of childhood tuberculous meningitis (TBM). Few studies have explored the correlation between these derangements and the staging of TBM disease (severity), or explored their role as biomarkers for vascular ischemic events, hydrocephalus, or seizures.

**Methods:** We aimed to identify the prevalence and the correlation between serum hyponatremia (mild, moderate and severe) and/or hypoglycorrhachia in relation to clinical TBM features such as stage of disease, seizures and stroke in children diagnosed with definite and probable TBM, between 1985 and 2015, at Tygerberg Hospital, Cape town, South Africa.

**Results:** The prevalence of hyponatremia was 344 out of 481 (71.5%) patients; 169 (49.1%) had mild hyponatremia, 146 (42.4%) moderate hyponatremia and 29 (8.4%) severe hyponatremia. Children with severe hyponatremia had higher frequency of stroke [odds ratio (OR) 4.36, 95% confidence interval (CI) 1.24–15.35; *p* = 0.01], brainstem dysfunction (OR 7.37, 95% CI 2.92–18.61; *p* < 0.01), cranial nerve palsies (OR 2.48, 95% CI 1.04–5.91; *p* = 0.04) and non-communicating hydrocephalus (OR 2.66, 95% CI 1.09–6.44; *p* = 0.03). Children with moderate hyponatremia and mild hyponatremia compared to those without hyponatremia similarly were more likely to exhibit signs of brainstem dysfunction (OR 1.91, 95% CI 1.11–3.28; *p* = 0.02) and hydrocephalus (OR 3.18, 95% CI 1.25–8.09; *p* = 0.01), respectively. On multivariable analysis only brainstem dysfunction was significantly associated with severe hyponatremia [adjusted odds ratio (aOR) 4.46, 95% CI 1.62–12.30; *p* < 0.01]. Children with hypoglycorrhachia compared to normoglycorrhachia were more likely to have had longer symptom duration prior to admission (OR 1.87, 95% CI 1.09–3.20; *p* = 0.02), non-communicating hydrocephalus (OR 1.64, 95% CI 0.99–2.71; *p* = 0.05), higher cerebrospinal white cell counts (OR 3.00, 95% CI 1.47–6.12; *p* < 0.01) and higher CSF protein concentrations (OR 2.51, 95% CI 1.49–4.20; *p* < 0.01). On multivariable analysis raised CSF protein concentration >1 g/L was significantly associated with hypoglycorrhachia (aOR 2.52, 95% CI 1.44–4.40; *p* < 0.01). Death rates did not differ by sodium level category or presence of hypoglycorrachia, however an increasing trend of children that had demised was noted the more severe the sodium category.

**Conclusion:** Hyponatremia and/or hypoglycorrhachia occur in more than two-thirds of children with TBM. Severe TBM disease complications such as brainstem dysfunction was associated with moderate hyponatremia, while severe hyponatremia was associated with brainstem dysfunction, stroke, cranial nerve palsies and non-communicating hydrocephalus. Cerebrospinal fluid (CSF) glucose depletion correlated with non-communicating hydrocephalus and increased CSF inflammation.

## Introduction

Worldwide, tuberculosis (TB) is one of the top 10 causes of death and the leading cause from a single infectious agent. Tuberculous meningitis (TBM) is estimated to represent 1% of all cases of TB, yet disproportionately contributes to TB-related morbidity and mortality (1). Young children, elderly patients and those with immunodeficiency, mostly HIV co-infection, are at increased risk of developing TBM, contributed to by malnutrition, anemia, advanced immune suppression and frequent TB exposure (2, 3). The peak age for pediatric TBM incidence is <5 years (4).

Hyponatremia has long been recognized as a serious potential metabolic consequence of TBM, occurring in 35–65% of children with the disease (5). It is independently associated with a poor outcome which has been attributed to either a more delayed presentation or increased severity of TBM disease (6). There are limited studies on TBM-associated hyponatremia, and the consequences thereof (7). A single study of 81 patients found that hyponatremia and polyuria was more severe and prolonged in patients with TBM-associated stroke, with it being postulated that hypovolemia and resultant hypoperfusion, together with TBM-associated vasculitis, contributes to infarction (7, 8).

Hypoglycorrhachia (abnormally low cerebrospinal fluid glucose concentration) has long been recognized as a predictor of central nervous system (CNS) microbial infection, including TBM in children. Hypoglycorrhachia is associated with higher degrees of CSF inflammation (9). The main driver of hypoglycorrhachia appears to be a combination of microbial meningitis with moderate to high degrees of CSF inflammation, suggesting that the presence of micro-organisms capable of catabolizing glucose is a determinant of hypoglycorrhachia. The elevated CSF lactate level that commonly accompanies a low CSF glucose concentration provides support for increased anaerobic metabolism contributing to these changes (10). Cerebral tissue is known to be extremely vulnerable to ischemic injury because of its relatively high oxygen consumption and near-total dependence on aerobic glucose metabolism. Literature on CSF glucose concentrations in ischemic stroke are lacking.

We aimed to describe the correlation between hyponatremia, hypoglycorrhachia, and the severity of TBM in children over a 30-year period.

## Methodology

### Study Population and Setting

This study was conducted at Tygerberg Hospital, the largest tertiary hospital in the Western Cape province of South Africa, serving as the academic and teaching hospital for Stellenbosch University. It lies within the metropole of Cape Town serving mostly poor communities in its geographical drainage area with a population ~2.6 million people. Children suspected of having TBM are referred from primary and regional hospitals to establish the diagnosis and for medical and surgical management of mainly neurological complications. In a minority of cases anti-tuberculous medication was initiated no longer than 48 h prior to transfer to Tygerberg Hospital.

Between 1985 and 2015, children were prospectively enrolled in several TBM research studies. Inclusion criteria for this current analysis were: (1) Age 3 months to 13 years; (2) “Definite” or “probable” TBM according to a uniform research case definition; (11) (3) Serum sodium samples at admission (for the serum sodium analysis); (4) CSF glucose samples at admission (for the CSF glucose analysis); (5) Baseline brain computed tomography (CT); (6) Written consent for the original prospective study from the caregiver with assent if the child was older than 7 years and competent to do so. Exclusion criteria were: (1) Age younger than 3 months or 13 years and older; (2) “Possible” or “No” TBM according to a uniform research case definition; (11) (3) Missing or incomplete data. The study was approved by the Human Research Ethics Committee of Stellenbosch University, South Africa (study nr. S20/05/112) and waiver of consent was approved.

### Clinical Procedures

On admission, all children underwent general physical and neurological examination in a dedicated pediatric neurology ward. Tuberculin skin testing was conducted as well as mycobacterial analysis of sputum, gastric washings and CSF. In patients with suspected raised intracranial pressure, brain CT was performed prior to lumbar puncture. All images were read during the admission period by experienced pediatric neurologists, followed by formal reporting by either a consultant radiologist or neuroradiologist. CSF analysis included biochemistry, microscopy after Ziehl-Neelsen staining and culture for *Mycobacterium tuberculosis by* inoculation of 0.5 ml CSF into BACTEC or Mycobateria Growth Indicator Tube (MGIT) automated liquid culture systems (Becton Dickinson Biosciences, Sparks, MD, USA). PCR-based assays were not routinely performed during the study period. HIV screening was performed in just under half of children as this was not a routine practice throughout the study period.

All children with TBM were treated with a short, intensified treatment regimen, isoniazid (20 mg/kg/day, maximum 400 mg daily), rifampin (20 mg/kg/day, maximum 600 mg daily), pyrazinamide (40 mg/kg/day maximum 2 g daily) and ethionamide (20 mg/kg/day, maximum 750 mg daily) for 6 months (12). All drugs were administered as a single daily dose before breakfast. Drugs were administered by nasogastric tube to children who were unable to swallow. Treatment was prolonged to 9 months in cases with HIV co-infection and/or isoniazid monoresistance. Corticosteroids (prednisone 2 mg/kg/day, maximum 60 mg) were prescribed for the first 4 weeks of therapy, followed by a 2-week tapering period, in all children with TBM (13).

### Case Definitions

TBM was defined as “definite” when CSF demonstrated acid-fast bacilli and/or positive *Mycobacterium tuberculosis* culture in a child with suggestive clinical presentation. “Probable” TBM was defined according to the uniform research case definition criteria, with points allocated for (a) clinical presentation, (b) CSF findings, (c) neuroimaging, (d) evidence of extra-neural TB, and (e) additional laboratory criteria (11). The refined British Medical Research Council (BMRC) criteria was used to classify TBM severity as follows: Stage I) Glasgow coma scale (GCS) of 15, without focal neurological deficit; Stage IIa) GCS of 15 with focal neurological deficit; Stage IIb) GCS of 11–14 with or without focal neurological deficit and Stage III) GCS <11 with or without focal neurological deficit (14, 15). Hemiparesis was clinically defined as unilaterally-reduced movement and skill in the face and/or upper and lower limb consistent with arterial ischemic infarction of the motor cortex and/or corticospinal tracts. Radiological arterial ischemic infarction was defined as neuroimaging evidence of infarction, i.e., interruption of blood flow eventually resulting in focal encephalomalacia. As radiological arterial ischemic infarction is not always demonstrated on early neuroimaging, and CT is not the optimal modality to detect small areas of arterial ischemic infarction in the territory of the middle cerebral artery perforators, we considered hemiparesis (uni- or bilateral) and/or radiological arterial ischemic infarction as evidence of stroke (16). Clinically raised intracranial pressure was defined as a bulging fontanelle, setting sun sign and/or acute onset strabismus in infancy, and papilledema in an older child. Brainstem dysfunction was defined as a constellation of signs and/or symptoms which included one or more of the following: marked depressed level of consciousness; impaired brainstem reflexes such as corneal, pupillary, dolls eye, oculovestibular, gag and cough; abnormal central breathing pattern; central autonomic instability and hemiplegia alternans.

Hyponatremia was defined as a serum sodium <135 mEq/L. Severity of hyponatremia was defined by the serum sodium level as follows: mild hyponatremia: serum concentration between 130 and 134 mEq/L; moderate hyponatremia: serum concentration between 120 and 129 mEq/L; severe hyponatremia: serum concentration <120 mEq/L (6). Moderately-raised CSF leucocyte count which is typical for TBM was defined as 10–500 cells/L (11). Normoglycorrhachia was defined as a CSF glucose concentration of either ≥2.2 mmol/L or a level which is ≥50% of the blood glucose and hypoglycorrhachia as a CSF glucose concentration of either <2.2 mmol/L or a level which is <50% of the blood glucose (9, 11).

### Statistical Analysis

Data analysis was performed using Statistical Package for the Social Sciences version 26 (SPSS Inc, Chicago, IL, USA). For descriptive purposes, frequencies were determined for categorical variables and medians and interquartile ranges (IQRs) for age and serum sodium (both non-parametrically distributed). Medians of independent samples (with non-parametric distribution) were compared, with the level of significance set at *p* < 0.05. Univariable logistic regression was used to determine odds ratios (ORs) and 95% confidence intervals (CIs) when comparing categorical variables between different categories of hyponatremia compared to normal serum sodium level, and hypoglycorrhachia to normoglycorrhachia, respectively. The level of significance was set at *p* < 0.05. Multivariable logistic regression analysis was performed, using all variables with a significance level of *p* < 0.10, in order to identify variables that were independently associated with different categories of hyponatremia compared to normal serum sodium level, and hypoglycorrhachia to normoglycorrhachia, respectively.

## Results

**Figure 1:**
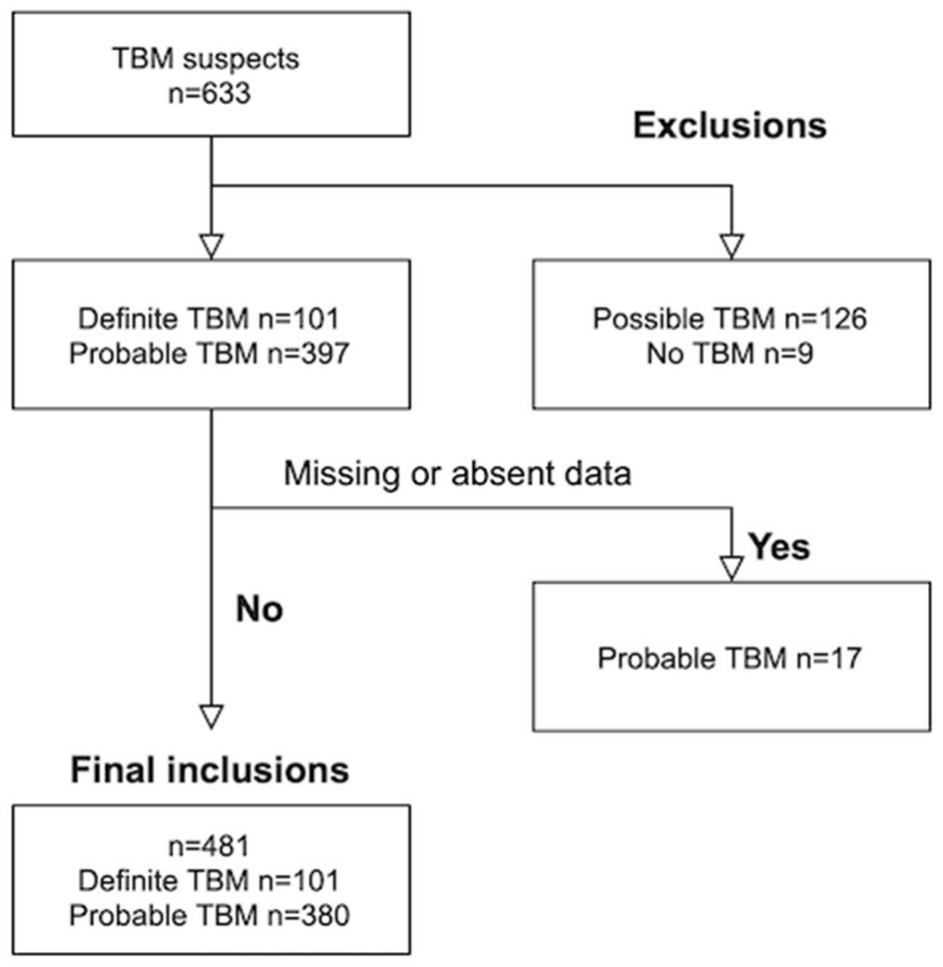
Patient flow.

Out of 633 TBM suspects, 153 were excluded; nine children with “No TBM,” 126 with “possible” TBM and 18 children with either missing serum sodium values at admission and/or absence of baseline CT brain imaging. Out of the 481 children with TBM that were included, 101 were diagnosed as “definite” TBM and 380 as “probable” TBM (see Figure 1). The median age was 28 months [interquartile range (IQR) 14–48.0] and 239 (49.7%) were male. The diagnosis of TBM was bacteriologically-confirmed in 91 (18.9%). TBM severity data was available in 454 children; stage I in 13 (2.9 %), stage IIa in 84 (18.5%), stage IIb in 124 (27.3%) and stage III in 233 (51.3%). HIV prevalence was 5.2% (12 out of 233 tested).

The prevalence of hyponatremia was 71.5% (344 out of 481 children); comprising 169 (49.1%) children with mild hyponatremia, 146 (42.4%) with moderate hyponatremia and 29 (8.4%) with severe hyponatremia. Ten children had hypernatremia (serum sodium >145 mmol/L). Table 1 shows the demographic, clinical, laboratory and radiologic parameters of children with and without serum hyponatremia (cohorted according to degree of severity).

**Table 1:** Clinical, laboratory and radiologic parameters of pediatric TBM with and without varying severity of serum hyponatremia.

		**All patients** **(*n* = 481)** *n*/*N* (%)^*^	**Without hyponatermia** **(*n* = 127)** ***n*/*N* (%)**	**Mild hyponatremia** **(*n* = 169)** ***n*/*N* (%)**	**Moderate hyponatremia** **(*n* = 146)** ***n*/*N* (%)**	**Severe hyponatremia** **(*n* = 29)** ***n*/*N* (%)**	**Comparison between** **hyponatremia categories**
							***p*** **-value**
Definite TBM		101/481 (21.0)	19/127 (15.0)	34/169 (20.1)	32/146 (21.9)	4/29 (13.8)	0.33
Male gender		239/481 (49.7)	57/127 (44.9)	82/169 (48.5)	80/146 (54.8)	15/29 (51.7)	0.76
Median age in months		28.0: IQR 14.0–48.0	23.0: IQR 12.0–48.0	30.0: IQR 16.0–49.0	29.0: IQR 16.0–49.0	28.0: IQR 24.0–44.5	0.94^#^
Vomiting		223/421 (53.0)	55/112 (49.1)	78/148 (52.7)	70/129 (54.3)	14/25 (56.0)	0.87
Fever		303/448 (67.6)	90/120 (75.0)	101/156 (64.7)	91/137 (66.4)	17/27 (63.0)	0.73
Seizures		193/421 (45.8)	49/112 (43.8)	67/148 (45.3)	59/129 (45.7)	13/25 (52.0)	0.57
Headache		107/421 (25.4)	28/112 (25.0)	40/148 (27.0)	34/129 (26.4)	5/25 (20.0)	0.46
Symptom duration > 5 days		401/481 (83.4)	111/127 (87.4)	138/169 (81.7)	120/146 (82.2)	24/29 (82.8)	0.94
HIV positive		12/233 (5.2)	3/71 (4.2)	4/83 (4.8)	3/62 (4.8)	2/12 (16.7)	0.14
Faltering weight gain or weight loss		208/477 (43.6)	49/119 (41.2)	76/157 (48.4)	57/136 (41.9)	10/27 (37.0)	0.69
GCS <15		357/454 (78.6)	90/119 (75.6)	125/160 (78.1)	109/139 (78.4)	24/27 (88.9)	0.20
TBM severity	Stage I	13/454 (2.9)	7/119 (5.5)	4/160 (2.5)	2/139 (1.4)	0	
	Stage IIa	84/454(18.5)	21/119 (17.6)	32/160 (20.0)	28/139 (20.1)	3/27 (11.1)	0.28
	Stage IIb	124/454 (27.3)	37/119 (31.1)	44/160 (27.5)	36/139 (25.9)	6/27 (22.2)	0.69
	Stage III	233/454 (51.3)	54/119 (45.4)	80/160 (50.0)	73/139 (52.5)	18/27 (66.7)	0.18
Stroke (hemiparesis and/or radiologic infarction)		301/445 (67.6)	77/119 (64.7)	108/160 (67.5)	92/139 (66.2)	24/27 (88.9)	**0.03**
Cranial nerve palsy		135/454 (29.7)	29/119 (24.4)	48/160 (30.0)	46/139 (33.1)	12/27 (44.4)	0.26
Raised ICP symptoms		98/453 (21.6)	28/119 (23.5)	31/160 (19.4)	30/138 (21.7)	7/27 (25.9)	0.63
Brainstem dysfunction		159/454 (35.0)	29/119 (24.4)	53/160 (33.1)	53/139 (38.1)	19/27 (70.4)	**<0.01**
Median serum sodium in mmol/L (IQR)		132.0 (127.0–135.0)					
CSF leucocytes 10–500 cells/L		402/454 (88.5)	107/119 (89.9)	146/160 (91.3)	121/139 (87.1)	23/27 (85.2)	0.79
CSF lymphocyte predominance		373/454 (82.2)	101/119 (84.9)	131/160 (81.9)	115/139 (82.7)	22/27 (81.5)	0.88
CSF protein concentration >1 g/L		334/449 (74.4)	85/116 (73.3)	109/159 (68.6)	114/138 (82.6)	19/27 (70.4)	0.15
CSF glucose <2.2 mmol/L or <50% of blood level		297/441 (67.3)	75/116 (64.7)	105/156 (67.3)	95/134 (70.9)	19/26 (73.1)	0.82
Basal meningeal enhancement		360/435 (82.8)	97/114 (85.1)	120/154 (77.9)	113/132 (85.6)	21/26 (80.8)	0.53
Tuberculoma (s)		60/435(13.8)	19/114 (16.7)	20/154 (13.0)	17/132 (12.9)	3/26 (11.5)	0.85
Hydrocephalus		400/435 (92.0)	99/114 (86.8)	147/154 (95.5)	122/132 (92.4)	24/26 (92.3)	0.98
Non-communicating hydrocephalus		116/494 (29.4)	25/97 (25.8)	40/145 (27.6)	43/122 (35.2)	8/23 (34.8)	0.11
Death		34/378 (9.0)	5/97 (5.2)	13/133 (9.8)	13/117 (11.1)	3/23 (13.0)	0.79

* *Includes patients with serum sodium >145 mmol/L.*

# *Comparison for mean age.*

*Zero stage I TBM with severe hyponatremia.*

*TBM, tuberculous meningitis; IQR, interquartile range; HIV, human immunodeficiency virus; GCS, glasgow coma scale; ICP, intracranial pressure; CSF, cerebrospinal fluid. The bold values reflect significant p value of ≤*.

**Figure 2:**
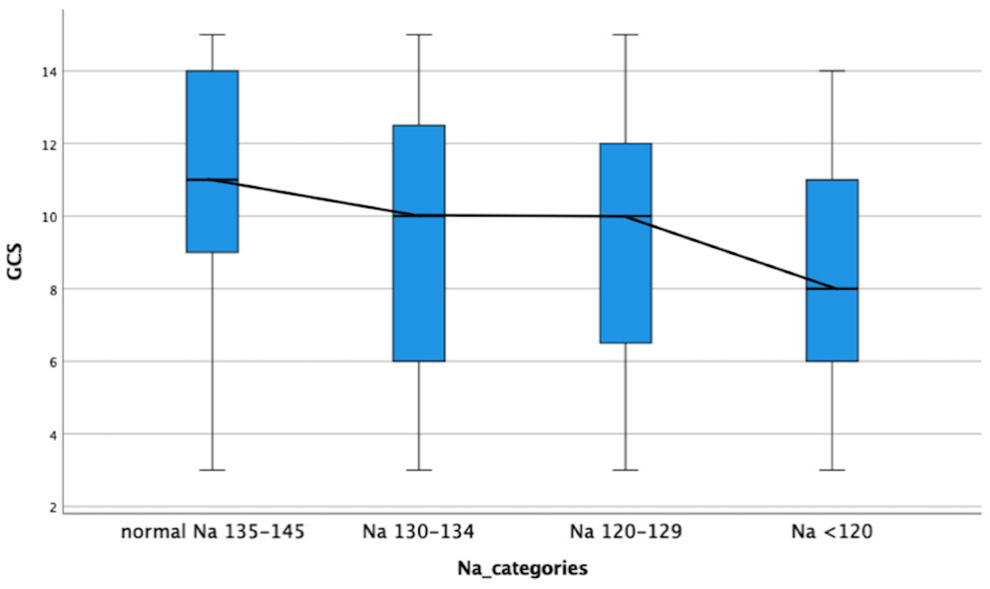
Decrease in median glasgow coma scale on admission with increased severity category serum hyponatremia in children with tuberculous meningitis. GCS, glasgow coma scale; Na, sodium.

**Figure 3:**
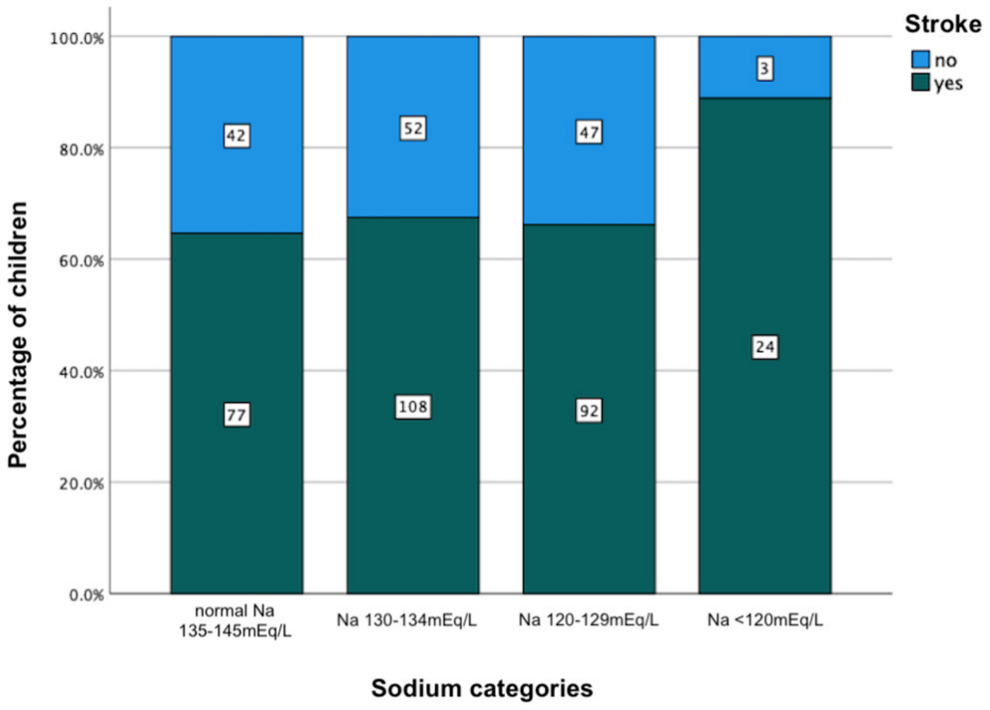
Increased percentage of stroke in relation to severity category of serum hyponatremia on admission in children with tuberculous meningitis. Na, sodium.

The comparison of demographic, clinical, laboratory and radiologic parameters between children without hyponatremia and children with varying severities of serum hyponatremia is shown in Table 2. Compared to children without hyponatremia, children with severe hyponatremia had higher frequency of stroke (clinically-persistent hemi- or quadriparesis with or without radiological arterial ischemic infarction) [odds ratio (OR) 4.36, 95% confidence interval (CI) 1.24–15.35; *p* = 0.01], brainstem dysfunction (OR 7.37, 95% CI 2.92–18.61; *p* < 0.01), cranial nerve palsies (OR 2.48, 95% CI 1.04–5.91; *p* = 0.04), and non-communicating hydrocephalus (OR 2.66, 95% CI 1.09–6.44; *p* = 0.03). Compared to children without hyponatremia, children with severe hyponatremia had shorter median symptom duration of 7.0 vs. 10.0 days; *p* = 0.03, however symptom duration was still prolonged in both groups (>5 days). Median symptom duration did not differ when comparing mild and moderate hyponatremia to children without hyponatremia. Children with moderate hyponatremia similarly were more likely to exhibit signs of brainstem dysfunction (OR 1.91, 95% CI 1.11–3.28; *p* = 0.02). Children with mild hyponatremia had higher frequency of hydrocephalus (OR 3.18, 95% CI 1.25–8.09; *p* = 0.01). On multivariable analysis of stage III TBM, cranial nerve palsy, brainstem dysfunction, stroke and non-communicating hydrocephalus in the categories of hyponatremia, only brainstem dysfunction was significantly associated with severe hyponatremia [adjusted odds ratio (aOR) 4.46, 95% CI 1.62–12.30; *p* < 0.01] (Table 2). Subgroup analysis of HIV positive children did not yield useful information as the group was too small, while subgroup analysis of the smaller definite TBM group only yielded a single significant finding; when comparing children with moderate hyponatremia to those without hyponatremia, they were more likely to present with cranial nerve palsies (OR 3.28, 95% CI 1.26–8.56; *p* = 0.01). Comparison of mild, moderate and severe hyponatremia with respect to demographic, clinical, laboratory and radiologic parameters are shown in Table 1; significant difference were observed for stroke and brainstem dysfunction. Figure 2 illustrates the decrease in median GCS on admission per worsening severity category of serum sodium, while Figure 3 illustrates the increased proportion of stroke in severe hyponatremia compared to other categories of serum sodium. No correlation was identified between serum sodium concentration and age of the child, symptoms (vomiting, fever, seizures, headache, weight loss), duration of symptoms, basal meningeal enhancement, presence of tuberculomas or cerebral infarction on CT imaging on admission. Although there was no statistical difference in children that had demised in the different categories of hyponatremia compared to normal serum sodium, there was an increasing trend as the hyponatremia severity category worsened [children with hyponatremia 5/97 (5.2%), mild hyponatremia 13/133 (9.8%), moderate hyponatremia 13/117 (11.1%), and severe hyponatremia 3/233 (13.0%)].

**Table 2:** Uni- and multivariable analysis of demographic, clinical, laboratory and radiologic parameters of TBM in patients with and without hyponatremia. Multivariable analysis.

	**Mild hyponatremia vs**.		**Moderate hyponatremia vs**.		**Severe hyponatremia vs**.	
	**normotraemia**		**normotraemia**		**normotraemia**	
	***p*-value**	**OR (95% CI)**	***p*-value**	**OR (95% CI)**	***p*-value**	**OR (95% CI)**
**Univariable analysis**						
Definite TBM	0.25	1.43 (0.77–2.65)	0.14	1.60 (0.85–2.98)	0.87	0.91 (0.28–2.91)
Male gender	0.50	1.17 (0.74–1.86)	0.10	1.49 (0.92–2.40)	0.51	1.32 (0.59–2.95)
Median age	0.49		0.33		0.92	
Vomiting	0.57	1.16 (0.71–1.89)	0.42	1.23 (0.74–2.04)	0.53	1.32 (0.55–3.16)
Fever	0.09	0.63 (0.37–1.07)	0.13	0.66 (0.38–1.14)	0.20	0.57 (0.23–1.37)
Convulsions	0.81	1.06 (0.65–1.74)	0.76	1.08 (0.65–1.80)	0.45	1.39 (0.58–3.32)
Symptom duration >5 days	0.13	0.60 (0.31–1.17)	0.18	0.62 (0.31–1.24)	0.44	0.65 (0.22–1.96)
Median symptom duration	0.42		0.12		**0.03**	
Weight faltering/loss	0.23	1.34 (0.83–2.17)	0.91	1.03 (0.63–1.70)	0.69	0.84 (0.36–1.99)
GCS <15	0.58	1.17 (0.67–2.05)	0.57	1.18 (0.66–2.11)	0.11	2.70 (0.76–9.58)
TBM Stage I	0.69	0.79 (0.24–2.60)	0.07	0.23 (0.05–1.14)	^*^	^*^
Stage IIa	0.65	1.12 (0.69–1.83)	0.77	1.10 (0.59–2.04)	0.30	0.52 (0.15–1.77)
Stage IIb	0.96	0.99 (0.64–1.53)	0.32	0.77 (0.45–1.30)	0.52	0.74 (0.29–1.87)
Stage III	0.86	0.97 (0.66–1.42)	0.17	1.40 (0.86–2.27)	0.09	2.04 (0.90–4.64)
Stroke	0.63	1.13 (0.69–1.87)	0.80	1.07 (0.64–1.79)	**0.01**	4.36 (1.24–15.35)
Cranial nerve palsy	0.30	1.33 (0.78–2.28)	0.12	1.54 (0.89–2.65)	**0.04**	2.48 (1.04–5.91)
Raised ICP	0.40	0.78 (0.44–1.39)	0.73	0.90 (0.50–1.62)	0.79	1.14 (0.44–2.97)
Brainstem dysfunction	0.11	1.54 (0.90–2.62)	**0.02**	1.91 (1.11–3.28)	**<0.01**	7.37 (2.92–18.61)
CSF leucocytes 10–500 cells/L	0.71	1.17 (0.52–2.63)	0.47	0.75 (0.35–1.64)	0.48	0.65 (0.19-2.18)
CSF lymphocyte predominance	0.51	0.81 (0.42–1.53)	0.64	0.85 (0.44–1.66)	0.66	0.78 (0.26–2.34)
CSF protein >1 g/L	0.40	0.80 (0.47–1.35)	0.07	1.73 (0.95–3.16)	0.76	0.87 (0.34–2.18)
CSF glucose <2.2 mmol/L or <50% of blood level	0.65	1.13 (0.68–1.87)	0.29	1.33 (0.78–2.27)	0.41	1.48 (0.58–3.82)
Basal meningeal enhancement	0.14	0.62 (0.33–1.17)	0.91	1.04 (0.51–2.12)	0.59	0.74 (0.24–2.22)
Tuberculoma (s)	0.40	0.75 (0.38–1.47)	0.40	0.74 (0.36–1.50)	0.52	0.65 (0.18–2.39)
Hydrocephalus	**0.01**	3.18 (1.25–8.09)	0.15	1.85 (0.80–4.29)	0.44	1.82 (0.39–8.49)
Non-communicating hydrocephalus	0.41	1.24 (0.74–2.08)	0.13	1.30 (1.57–2.82)	**0.03**	2.66 (1.09–6.44)
Death	0.20	1.99 (0.69–5.79)	0.12	2.30 (0.79–6.70)	0.17	2.76 (0.61–12.50)
	***p*** **–value**	**aOR (95% CI)**	***p*** **-value**	**aOR (95% CI)**	***p*** **-value**	**aOR (95% CI)**
**Multivariable analysis**						
TBM stage III	0.64	0.89 (0.55–1.44)	0.64	1.13 (0.68–1.86)	0.37	0.63 (0.22–1.74)
Cranial nerve palsy	0.86	0.96 (0.62–1.50)	0.41	1.21 (0.76–1.92)	0.56	1.30 (0.55–3.05)
Brainstem dysfunction	0.63	0.89 (0.56–1.43)	0.39	1.24 (0.76–2.02)	<0.01	4.46 (1.62–12.30)
Stroke	0.91	0.96 (0.65–1.63)	0.48	0.84 (0.52–1.36)	0.13	1.93 (0.82–4.54)
Non–communicating hydrocephalus	0.86	1.04 (0.68–1.59)	0.94	0.98 (0.63–1.53)	0.17	2.49 (0.68–9.15)

*TBM, tuberculous meningitis; OR, odds ratio; CI, confidence interval; GCS, glasgow coma scale; ICP, intracranial pressure; CSF, cerebrospinal fluid; aOR, adjusted odds ratio.*

* *Zero stage I TBM with severe hyponatremia. The bold values reflect significant p value of ≤*.

Table 3 reflects the demographic, clinical, laboratory and radiologic parameters of children with and without CSF hypoglycorrhachia. The prevalence of hypoglycorrhachia was 72.9% (306 out of 420). The comparison of demographic, clinical, laboratory and radiologic parameters in children with and without hypoglycorrhachia are shown in Table 4. Children with hypoglycorrhachia were more likely to exhibit longer symptom duration (OR 1.87, 95% CI 1.09–3.20; *p* = 0.02) and non-communicating hydrocephalus (OR 1.64, 95% CI 0.99–2.71; *p* = 0.05) than children with normal CSF glucose levels. In addition, more children with hypoglycorrhachia had moderately-raised CSF white cell count (OR 3.00, 95% CI 1.47–6.12; *p* < 0.01) and raised CSF protein concentration >1 g/L (OR 2.51, 95% CI 1.49–4.20; *p* < 0.01), typical of TBM, than those with normal CSF glucose. On multivariable analysis of long symptom duration >5 days, moderately-raised CSF white cell count, raised CSF protein concentration >1 g/L and non-communicating hydrocephalus, only raised CSF protein concentration >1 g/L was significantly associated with hypoglycorrhachia (aOR 2.52, 95% CI 1.44–4.40; *p* < 0.01) (Table 4). No correlation was identified between hypoglycorrhachia and age, symptoms (vomiting, fever, seizures, headache, and weight loss), basal meningeal enhancement, presence of tuberculomas or stroke (clinically persistent hemi- or quadriparesis with or without radiological arterial ischemic infarction).

**Table 3:** Demographic, clinical, laboratory and neuroimaging features in pediatric TBM with and without hypoglycorrhachia 1985–2015.

		**Normoglycorrhacia** **(*n* = 114) *n*/*N* (%)**	**Hypoglycorrhacia** **(*n* = 306) *n*/*N* (%)**
Definite TBM		21/114 (18.4)	60/306 (19.6)
Male gender		60/114 (52.6)	153/306 (50.0)
Median age in months		29.0: IQR 14.5–63.5	27.0: IQR 15.0–47.0
Vomiting		46/100 (46.0)	146/267 (54.7)
Fever		70/107 (65.4)	192/284 (67.6)
Convulsions		47/100 (47.0)	122/267 (45.7)
Headache		28/100 (29.0)	63/267 (23.6)
Symptom duration >5 days		86/113 (76.1)	262/306 (85.6)
HIV positive		6/58 (10.3)	3/129 (2.3)
Faltering weight gain or weight loss		41/107 (38.3)	133/285 (46.7)
GCS <15		86/114 (75.4)	236/300 (78.7)
TBM Stage	Stage I	6/114 (5.3)	6/300 (2.0)
	Stage IIa	22/114 (19.3)	58/300 (19.3)
	Stage IIb	29/114 (25.4)	91/300 (30.3)
	Stage III	57/114 (50.0)	145/300 (48.3)
Stroke (hemiparesis and/or radiologic infarction)		66/107 (61.7)	200/287 (69.7)
Cranial nerve palsy		27/107 (25.2)	85/287 (29.6)
Raised ICP		26/107 (24.3)	58/286 (20.3)
Brainstem dysfunction		33/107 (30.8)	100/287 (34.8)
Median serum sodium in mmol/L (IQR)		132.0 (127.5–136.0)	131.0 (127.0–135.0)
CSF leucocytes 10–500 cells/L		90/107 (84.1)	270/287 (94.1)
CSF lymphocyte predominance		88/107 (82.2)	238/287 (82.9)
CSF protein concentration >1 g/L)		73/107 (68.2)	242/287 (84.3)
Basal meningeal enhancement		84/106 (79.2)	230/275 (83.6)
Tuberculoma (s)		16/106 (15.1)	35/275 (12.7)
Hydrocephalus		94/106 (88.7)	255/275 (92.7)
Non-communicating hydrocephalus		27/100 (27.0)	103/273 (37.7)
Death		8/89 (9.0)	23/236 (9.7)

*TBM, tuberculous meningitis; IQR, interquartile range; GCS, glasgow coma scale; ICP, intracranial pressure; CSF, cerebrospinal fluid*.

**Table 4:** Uni- and multivariable analysis of demographic, clinical, laboratory and radiologic parameters in pediatric TBM with and without hypoglycorrhachia.

**Univariable analysis**		
	***p*-value**	**OR (95% CI)**
Definite TBM	0.78	1.08 (0.62–1.87)
Male	0.57	0.88 (0.57–1.36)
Median age	**0.35**	
Vomiting	0.14	1.42 (0.89–2.27)
Fever	0.62	1.13 (0.70–1.81)
Convulsions	0.82	0.95 (0.60–1.50)
Symptom duration >5 days	**0.02**	1.87 (1.09–3.20)
Median symptom duration	0.53	
Weight faltering/loss	0.14	1.41 (0.90–2.22)
GCS <15	0.48	1.20 (0.72–2.00)
TBM Stage I	0.09	0.37 (0.12–1.16)
Stage IIa	0.99	1.00 (0.58–1.73)
Stage IIb	0.33	1.28 (0.78–2.08)
Stage III	0.76	0.94 (0.61–1.44)
Stroke (hemiparesis and/or radiologic infarction)	0.13	1.43 (0.90–2.27)
Cranial nerve palsy	0.39	1.25 (0.75–2.07)
Raised ICP	0.39	0.79 (0.47–1.34)
Brainstem dysfunction	0.46	1.20 (0.74–1.93)
Median serum sodium	0.32	
CSF leucocytes 10–500 cells/L	**<0.01**	3.00 (1.47–6.12)
CSF lymphocyte predominance	0.87	1.05 (0.59–1.88)
CSF protein >1 g/L	**<0.01**	2.51 (1.49–4.20)
Basal meningeal enhancement	0.31	1.34 (0.76–2.36)
Tuberculoma (s)	0.54	0.82 (0.43–1.55)
Hydrocephalus	0.20	1.63 (0.77–3.46)
Non-communicating hydrocephalus	**0.05**	1.64 (0.99–2.71)
Death	0.84	1.09 (0.47–5.54)
**Multivariable analysis**		
	***p*** **-value**	**aOR (95% CI)**
Symptom duration >5 days	0.12	1.61 (0.89–2.93)
CSF leucocytes 10–500 cells/L	0.06	2.21 (0.98–5.01)
CSF protein >1 g/L	<0.01	2.52 (1.44–4.40)
Non-communicating hydrocephalus	0.10	1.58 (0.92–2.71)

*TBM, tuberculous meningitis; OR, odds ratio; CI, confidence interval; ICP, intracranial pressure; CSF, cerebrospinal fluid; aOR, adjusted odds ratio. The bold values reflect significant p value of ≤*.

## Discussion

More than two-thirds of children with TBM in this study exhibited hyponatremia, which is higher than the proportion reported in other studies (5). The severity of the serum hyponatremia appears to be of clinical importance as children with TBM and severe hyponatremia were more likely to exhibit severe neurological stigmata such as signs of brainstem dysfunction, cranial nerve palsies, stroke and non-communicating hydrocephalus. In this context, it is important to appreciate that the signs and symptoms of raised intracranial pressure (ICP) and brainstem dysfunction often overlap. Brainstem injury, for example, may mimic all the signs of raised ICP whilst both raised ICP and brainstem dysfunction can induce cranial neuropathies. Serum hyponatremia itself may contribute to raised ICP and poor outcome through worsening of cerebral edema.

The syndrome of inappropriate antidiuretic hormone (SIADH) and cerebral salt wasting syndrome (CSWS) have been implicated in the development of hyponatremia in TBM. Previously, SIADH was assumed to be the commonest cause of hyponatremia in TBM, however more recent studies report CSWS as more common (7). The differentiation between the two conditions is often difficult in resource-constrained settings, however the management of serum hyponatremia secondary to intracranial causes is uniform irrespective of etiology. Fluid restriction may be harmful in TBM, as it may potentially compromise cerebral blood flow and induce cerebrovascular thrombosis, therefore the treatment of intracranial-related hyponatremia should be hypertonic saline in all cases. We postulate that the increased risk of stroke in the children with TBM and severe hyponatremia can be attributed to CSWS-induced volume contraction, supported by a study from Misra and colleagues who reported that hypovolemia due to CSWS may contribute to stroke in TBM (8). Similarly, the correlation between severe hyponatremia and hydrocephalus can be explained by excessive brain natriuretic peptide (BNP) release secondary to ventricular distention.

A reduction in CSF glucose concentration and a change in the CSF: serum glucose ratio is often used as one of the diagnostic indicators of meningitis. Glucose is mainly transported into the CSF by facilitation across the choroid plexus and the ventricular and subarachnoid capillary system while some of it is transported by simple diffusion. Current theories postulate that the pathogenesis of hypoglycorrhachia is multifactorial and includes inhibition of glucose entry into the subarachnoid spaces due to alterations in the blood brain barrier, increased rate of glucose transport across the arachnoid villi, increased glycolysis by leucocytes and bacteria and increased rate of metabolism in the brain and spinal cord. Of interest is that in this study children with TBM and hypoglycorrhachia exhibited higher CSF cell counts and protein than those with normal glucose concentrations; a finding that can be attributed to the higher degrees of CSF inflammation (9). Few studies have explored the clinical significance of hypoglycorrhachia in children with TBM at onset of disease. In this study, children with TBM and hypoglycorrhachia were more likely to exhibit longer symptom duration, as well as non-communicating hydrocephalus. The former can be explained by exposure to a longer period of inflammation, whilst the latter is postulated to be due to association with more severe inflammation, increasing the risk of obstruction of CSF flow.

Strengths of this study included a very large sample size collected prospectively over a 30-year period. The inclusion, exclusion and diagnostic criteria were well-defined. Study limitations include incomplete data relating to some laboratory parameters and incomplete HIV testing. HIV testing only became routinely available during the latter part of the study and in some of the children the HIV status was unknown. Another study limitation is the lack of analysis comparing sodium as a continuous variable with various categorical outcomes, taking into account possible confounders, potentially yielding interesting findings. The etiology of the serum hyponatremia was not determined as urine osmolality and sodium were not routinely requested. We were thus unable to distinguish between CSWS and SIADH. Despite this, it is important to note that the management of serum hyponatremia secondary to intracranial causes is uniform, irrespective of etiology. CT imaging is often the only imaging modality that is readily available in resource-constrained TB endemic settings. Unfortunately, CT is less sensitive at detection of acute arterial ischemic lesions (compared to MRI) which may have resulted in an underestimation of the number of strokes. Further limitations were that we were unable to localize the site of infarction or quantify the degree of basal enhancement on CT brain. For these reasons we also deemed persistent uni- or bilateral hemiplegia (in the absence of TB mass lesions and Todd's paresis) as manifestations of stroke. Data on cause of demise was not available and is an additional study limitation.

## Conclusion

Hyponatremia and/or hypoglycorrhachia occur in more than two-thirds of children with TBM. Severe TBM disease complications such as brainstem dysfunction was associated with moderate hyponatremia, while severe hyponatremia was associated with brainstem dysfunction, stroke, cranial nerve palsies and non-communicating hydrocephalus. Increased stroke risk in children with TBM and severe hyponatremia may be attributed to CSWS-induced volume contraction. Children with TBM and hypoglycorrhachia had higher CSF cell counts and protein; a finding that could be attributed to higher degrees of CSF inflammation. Future research is encouraged to affirm these findings.

## Data Availability Statement

The data analyzed in this study is subject to the following licenses/restrictions: The dataset is electronically captured on RedCap and is subject to ongoing research on pediatric TBM, approved by the Human Research Ethics Committee of Stellenbosch University, South Africa. Requests to access these datasets should be directed to Regan S. Solomons, regan@sun.ac.za.

## Ethics Statement

The studies involving human participants were reviewed and approved by Human Research Ethics Committee of Stellenbosch University, South Africa (study nr. S20/05/112). Written informed consent to participate in this study was provided by the participants' legal guardian/next of kin.

## Author Contributions

RS and RSo contributed to conception and design of the study, analyzed, and interpreted data. RS, RSo, RT, and JS wrote and revised sections of the manuscript and critically reviewed the manuscript. All authors contributed to the article and approved the submitted version.

## Funding

RSo was supported by the National Research Foundation of South Africa (109437). JS was supported by a Clinician Scientist Fellowship jointly funded by the UK Medical Research Council (MRC) and the UK Department for International Development (DFID) under the MRC/DFID Concordat agreement (MR/R007942/1).

## Author Disclaimer

The content hereof is the sole responsibility of the authors and does not necessarily represent the official views of the funders.

## Conflict of Interest

The authors declare that the research was conducted in the absence of any commercial or financial relationships that could be construed as a potential conflict of interest. The reviewer NB declared a past co-authorship with some of the authors RT, JS, and RSo to the handling editor.

## Publisher's Note

All claims expressed in this article are solely those of the authors and do not necessarily represent those of their affiliated organizations, or those of the publisher, the editors and the reviewers. Any product that may be evaluated in this article, or claim that may be made by its manufacturer, is not guaranteed or endorsed by the publisher.
